# The Pathology of Rheumatic Facial Paralysis

**Published:** 1877-08

**Authors:** O. Berger


### Translations.

### THE PATHOLOGY OF RHEUMATIC FACIAL PARALYSIS.

By Dr. O. Berger.

Translated from the *Deutsche Med. Wochenschrift*, Dec. 9, 1876.

By H. M. Bannister, M. D.

The extraordinary frequency of rheumatic facial paralysis, and in so many cases the indisputable clearness of its etiological conditions, very naturally cause it to be considered, as has been usually the case, the typical form of rheumatic nervous paralysis. Facial paralysis itself has been the starting point of a series of investigations, which the pathology of peripheral paralysis has to thank for the very high grade of perfection the diagnosis and prognosis of these affections have attained. Still, there remains a very palpable defect to be remedied, pathological anatomy is not in the condition to inform us as to the nature and seat of the anatomical lesion underlying facial paralysis. There remains to be made, therefore, only a careful clinical analysis, to supply the decisive answer to the question of the localization of the cause of the paralysis. Whoever is accustomed, not to notice merely the gross paralysis of the facial muscles of expression, but, mindful of the important physiological routes of conduction included in the facial nerve, to take the trouble to notice also other functional disturbances, will easily be in a position to give a tolerable local diagnosis. Erb* has recently reviewed the subject in an excellent memoir, and has demonstrated that with especial attention to the separate branches of the facial nerve, (posterior auricular, chorda tympani, stapedius, superficial petrosal nerve,) an absolute localization of the anatomical seat is possible in many cases. The fact that rheumatic facial para-

*On Rheumatic Facial Paralysis. *Deutsche Archiv. f. Klin. Medicin* 15 Bd.

lysis regularly affects all the facial branches, makes permissible the conclusion that the lesion which affects the conduction of the nerve is situated in the stem of the facial and not in any one of its branches. This can be either in the short section outside of the Fallopian canal, (after its exit from the stylo-mastoid foramen), within the same, or in the course of the nerve up to the base of the cranium. Trouble in this last named situation will scarcely be looked for, and in general we have to do with disorder in the first mentioned two localities. Various authors have expressed the opinion that every case of rheumatic paralysis, the light as well as the severer forms, has its anatomical seat in the Fallopian canal. Thus Baerwinkel* sometime since, and again more recently, has stated that the disease is always accompanied with an affection of the middle ear, the lighter ones with a serous, quickly absorbable exudation, and the severer forms with a plastic exudation. Convincing grounds for this were not given, and on the other hand the view is sufficiently satisfactory that in mild cases the anatomical lesion is situated in the nerve stem outside the Fallopian canal, and in severe ones within the same, and it has received the most general adoption.† In the inflammatory (rheumatic) swelling of the neurilemma which is usually considered to be the anatomical basis of the "refrigatory" facial paralysis, there would be in the first case, with its favorable mechanical conditions, only a mild form of the paralysis, and in the second, on the other hand, on account of limitation of space, a severe compression, and resulting secondary degeneration of the nerve would be produced. Erb, in his conclusions in the above mentioned extended memoir, speaks in favor of this view. As characteristic of the localization of the lesion in the stem of the facial after its exit from the stylo-mastoid foramen, we may mention the paralysis of the facial branches while the hearing, taste, salivary secretion, and the palate remain unaffected. A further evidence of this localization is an

---

**Archiv. der Heilkunde*, 1867, *S.* 82. *Deutsche Arch. f. Klin. Med.* 14 Bd. S. 122.

†Compare, among others, the exposition of facial paralysis by Eulenberg (Lehrbuch der funct. Krankh.) and by Erb, (Ziemssen's Handbuch der spec. Pathol.).

intact condition of the posterior auricular nerve, which leaves the stem close to the stylo-mastoid foramen, and innervates the occipital and the small aural muscles, and which, as Erb remarks, forms the boundary mark for the portions of the facial within and without the Fallopian canal. In spite of the abundance of casuistic material there are few cases in which this criterion can be made use of. The reason for this is sufficiently plain. The paralysis of this branch can only exceptionally be determined by the failure to move the ear, in those rare individuals who possess the ability to do so normally. Nevertheless, we can resort to another means to accomplish this. The electrical examination should be resorted to in all these cases, and it would not be difficult to demonstrate the pathological condition of this nerve. Thus Erb says that he himself in all severe cases in which he suspected the implication of this nerve has found it upon applying the electrical tests, a statement that perfectly coincides with my own experience. The case is different, however, with those slighter forms in which we find no perceptible change in the electric excitability, and in which we also desire to test the mobility. Erb has therefore suggested, correctly, that it would be to the highest degree interesting to obtain some certain data upon the condition of the posterior auricular nerve in the milder cases of rheumatic facial paralysis. I am now able to satisfy this want. For some years I have maintained in my lectures the proposition, supported by numerous clinical demonstrations, that the anatomical localization in rheumatic facial paralysis, in the mild as well as in the severer forms, must always be sought for in the Fallopian canal; and that the position assigned the lesion by the majority of authors, in the short section of the nerve after its exit from the stylo-mastoid foramen, is perfectly unsupported by evidence Thus it is incomprehensible why injurious atmospheric influences should always affect the main stem of the nerve and never its separate branches, notwithstanding the fact that the latter are relatively much the most exposed. I know of no case of recognizable rheumatic facial paralysis in which all the facial muscles of the side were not involved in the paralysis, and must refer opposing statements to fallacious observations. A relative integrity of certain branches may indeed be observed under peculiar circumstances,* but never an absolutely intact condition of single muscles. There is, moreover, no positive proof for the position I have above mentioned. The grounds upon which this statement seems to me to rest are as follows:

1. I can enumerate a number of cases of slight (rheumatic) facial paralysis in which electrical examination a short time after the appearance of the paralysis (8 to 80 hours), showed a perceptible and indubitably proven increase of the direct and indirect electrical irritability (to both currents). In all these cases the posterior auricular nerve and its innervated muscular region shared in this increase. I have been able repeatedly to demonstrate this to my hearers.

2. Several cases in which the disturbances of function on the part of the chorda tympani, twice also of the stapedius, allowed a diagnosis of the lesion within the Fallopian canal, nevertheless followed the course of the milder forms, and during their whole course showed no indication of the "*Entartungsreaction*," the paralysis disappearing in from eighteen to twenty-seven days.

3. Finally, I have come across one case that is absolutely confirmatory of my view. A few words will suffice for its description. On October 12th of this year, my colleague, Dr. Hannes, referred to me a railroad employe, August Schikora, aged thirty-eight, for electrical treatment. Three days previously he was taken with facial paralysis, from working many hours with heated sweating body in a draft of air under an open shed. No disturbances of sensibility or aural symptoms had appeared. On the contrary, the patient stated that since the appearance of the paralysis, while eating he had the sensation on the right anterior half of the tongue as if every thing was unsalted, but still

*A single instance only is reported recently by Baerwinkel, of uninvolved posterior auricular nerve, in a case of severe paralysis with proven localization within the Fallopian canal, (chorda tympani implicated). It cannot be accepted as a satisfactory case of "isolated exemption," since, according to the author's own statement, the faradic reaction (and as it appears, the motility also) was diminished as compared with that of the sound side.

there were no spontaneous subjective taste sensations. This peculiarity, moreover, disappeared entirely in a few days. *Status præsens*, October 12. Total right facial paralysis. Uvula and palate normal, alike when at rest and in movement. With objective testing of the sense of taste, the same perfection was found on the right as on the sound side. No abnormality of the salivary secretion. Tongue projected straight forward; no disorder of hearing. Sensibility altogether intact, reflex excitability of the paralyzed muscles increased.

*Electrical examination.* High degree of increase of faradic and galvanic (direct and indirect) excitability, tolerably uniform in all branches, and especially demonstrable in the ramus frontalis; difference of the faradic contraction minimum 15–20 mm., with stronger currents the right contraction somewhat stronger than the left. No qualitative alteration of the contraction law; on the other hand, a notable increase of the mechanical irritability, demonstrable from the separate nerve branches as well as by direct excitation of the muscle; no retarded character of the contraction. The posterior auricular nerve and its muscles (occipital, retrahens auri, and attollens auri) also showed this increased reaction. While I was already clear in mind as to the participation in the paralysis of the posterior auricular nerve and its muscles, this fact was, in this case, also demonstrable by testing the voluntary action of these muscles. In reply to questions, the patient stated that he had been conscious immediately after the occurrence of the paralysis, together with the general loss of motility of the muscles of the face, of an inability to do what he had long been able to do, to move the ear on the right side.* Even the first days following the

*This ability seems, according to my private experience, to be not altogether so uncommon. Yet it rarely happens that a person thus gifted suffers from facial paralysis. Two analogous cases, previously seen by me were of the severer variety and unsuited for the decision of the matter in question. It is self-evident that the independence of the contraction must be supported by close examination and palpation of the relatively strongly developed ear muscles, and of the adjoining parts of the head. By energetic innervation of the frontal muscles it is tolerably easy to produce a pseudo-movement of the concha.

paralysis he had proven this to himself and to his wife before the mirror. Examination revealed that the right ear like the facial muscles remained perfectly quiet to all voluntary impulses, while the left could be moved forwards and backwards voluntarily in the most pronounced manner. On the fifteenth of October (sixth day of the paralysis) the electric irritability had already become less from day to day, and on the twentieth it was exactly alike on either side of the face; the posterior auricular also participated in this change. From the commencement of the treatment the paralysis visibly improved, and the mobility of the right ear increased in the same ratio as the return of mobility in the muscles of expression, it was interesting to notice its gradual improvement. On the thirtieth of October (twenty-first day after the appearance of the paralysis) I was able to discharge the patient thoroughly cured without a trace of asymmetry; and with a special subject of enjoyment on his part, that with the perfect functional ability of his facial muscles, he had also recovered his old power over his right ear.

This case affords a characteristic example of the lighter form of rheumatic facial paralysis. On the fifth day there were signs of returning nervous conduction, a week later the motility had perceptibly improved, and after three weeks—even a day or two sooner—the cure was complete. The participation of the posterior auricular nerve, the primary increase of electric excitability, and its later decrease, show that the anatomical seat of the lesion is to be sought within the Fallopian canal. Immovability of the corresponding ear, definitely established, and a localization of the lesion in the trunk of the nerve after its exit from the stylo-mastoid foramen, together, are incompatible.[7]

From the normal condition of sense of taste and the salivary secretion (the slight temporary paraesthesia of taste must be referred to a slight irritation of the chorda tympani by reason of its neighborhood to the lesion), we are led in one case to place the special seat in the lowest portion of the Fallopian canal, external to the giving off of the chorda.

Thus we have afforded the desired evidence of the localization of the lesion interrupting nervous conduction in the Fallopian canal, in the milder form of rheumatic facial paralysis. So long as the opposed condition (absolute freedom from paralysis of the ear muscles) is not proven, the presumption of the localization of the lesion outside of the bony canal appears to be a gratuitous and unsupported hypothesis.*

Under any circumstances, our observation is an unassailable proof that the difference of the affection is not due to a different location, but to the intensity of the anatomical process; it is not—as according to the views generally held hitherto—the indications for one or another seat of the anatomical lesion, but singly and alone a methodic electrical examination—and this some days after the appearance of the paralysis—that gives us a satisfactory criterion for a reasonable prognosis in regard to the duration of the not dangerous, it is true, but nevertheless very disagreeable rheumatic facial paralysis.

*Here comes in the question, why the injurious local influence of cold never affects single branches, but always the nerve stem? Perhaps we may mention here those cases of slight local rheumatic affections, not infrequently met with, in which from exposure to cold air, drafts, etc. of the face, there is developed a certain stiffness and sense of immobility—generally in the muscles of the cheek—which lasts but a short time and then disappears. Here we have perhaps to do with slight disturbances of nutrition of certain nerve-twigs, which on account of the unrigid character of the soft surrounding tissues do not give rise to any compression interrupting nervous conduction. We may also mention the slight disturbance of sensibility almost always accompanying these conditions, which must naturally seem to us to be due to the intimate association of the trigeminus fibres with the branches of the anserine plexus; while in the case of a lesion of the main trunk of the facial the trigeminus branches are naturally unimplicated.

